# Synthesis, Characterization and Testing of Antimicrobial Activity of Composites of Unsaturated Polyester Resins with Wood Flour and Silver Nanoparticles

**DOI:** 10.3390/ma14051122

**Published:** 2021-02-27

**Authors:** Przemysław Pączkowski, Andrzej Puszka, Malgorzata Miazga-Karska, Grażyna Ginalska, Barbara Gawdzik

**Affiliations:** 1 Department of Polymer Chemistry, Faculty of Chemistry, Institute of Chemical Sciences, Maria Curie-Sklodowska University in Lublin, Gliniana 33, 20-614 Lublin, Poland; andrzej.puszka@umcs.pl (A.P.); barbara.gawdzik@umcs.pl (B.G.); 2 Chair and Department of Biochemistry and Biotechnology, Faculty of Pharmacy, Medical University of Lublin, Chodzki 1, 20-093 Lublin, Poland; malgorzata.miazga-karska@umlub.pl (M.M.-K.); grazyna.ginalska@umlub.pl (G.G.)

**Keywords:** antibacterial activity, wood–resin composites, unsaturated polyester resin, recycled PET, wood flour, renewable resources, silver nanoparticles

## Abstract

This paper presents the properties of the wood-resin composites. For improving their antibacterial character, silver nanoparticles were incorporated into their structures. The properties of the obtained materials were analyzed in vitro for their anti-biofilm potency in contact with aerobic Gram-positive *Staphylococcus aureus* and *Staphylococcus epidermidis*; and aerobic Gram-negative *Escherichia coli* and *Pseudomonas aeruginosa*. These pathogens are responsible for various infections, including those associated with healthcare. The effect of silver nanoparticles incorporation on mechanical and thermomechanical properties as well as gloss were investigated for the samples of composites before and after accelerating aging tests. The results show that bacteria can colonize in various wrinkles and cracks on the composites with wood flour but also the surface of the cross-linked unsaturated polyester resin. The addition of nanosilver causes the death of bacteria. It also positively influences mechanical and thermomechanical properties as well as gloss of the resin.

## 1. Introduction

All the possibilities of using new products, including composites with the addition of recycled wood, help to reduce the environmental impact and consumption of conventional polymers. Among the various synthetic polymers, unsaturated polyester resins are the most commonly known in preparing composites and exceed 80% of all components [1,2]. The global unsaturated polyester resin market will grow at a steady 5.3% CAGR over the forecast period (2019–2029) [3,4,5]. Greater concern about health as a result of industrial activities enhances the demand for unsaturated polyester resins. Modern products use a low content of styrene, which reduces the harmful effects of workmen’s exposure to poisonous gases, and thus decreases carbon dioxide emissions improving safety standards in many industries [6]. Such materials are widely applied in the production of yachts, kayaks, sailboats, bathtubs, shower cabins, etc., as well as housing goods and medical equipment. 

It is known that microorganisms survive on inanimate “touch” surfaces for a long time. This can be particularly troublesome in health care where patient immunity is at a greater risk of infections. Touch surfaces in hospital rooms can serve as a source or a reservoir of bacteria expansion.

A nosocomial infection can be contracted in various ways in hospitals, nursing homes, rehabilitation centers, clinics and even diagnostic laboratories [7,8,9]. Besides contaminated equipment, bed sheets or air droplets, the infection is also spread by medical personnel themselves. In some cases, the microorganisms may originate from the patient’s own skin and become opportunistic after surgery or other procedures that compromise the skin protective barrier. Bacteria can enter the bloodstream as a serious complication of infection during surgery or because of catheters and other foreign bodies entering the arteries or veins.

The common pathogens found in the healthcare settings are *Escherichia coli (E. coli)* and *Pseudomonas aeruginosa (P. aeruginosa)* Gram-negative and rod-shaped bacteria, while *Staphylococcus aureus (S. aureus)* and *Staphylococcus epidermidis (S. epidermidis)* are round Gram-positive bacteria.

One of the most important and common species of Gram-positive bacteria is *Staphylococcus*. These bacteria are normally found on the skin or in the digestive tract and can enter the bloodstream, where *S. aureus* is responsible for the most common healthcare-associated infections. 

As a result of infections in the respiratory tract, the genitourinary system, the gastrointestinal tract or the hepatobiliary system, Gram-negative bacteria are able to enter the bloodstream. This type of Gram-negative bacteremia is more commonly associated with the elderly population, where *E. coli* is one of the most common reasons of its formation.

A number of compounds added to materials can reduce the risk of bacteria growing on the surface. The use of silver or copper nanoparticles causes the antimicrobial properties of biocomposites [10,11,12]. An alternative approach to reducing healthcare-associated pathogens is the use of ultraviolet irradiation [13,14,15,16]. Silver nanoparticles (AgNPs) are an important component of nanomaterials in a wide range of industrial and medical applications. There are official EU regulations that allow the use of nanosilver [17]. In another regulation there is the conclusion about the safety of colloidal silver in nano form only at low concentrations [18]. 

Unfortunately, nanosilver can be risky to human health. According to Ahamed et al. [19], AgNPs produce toxicity that targets various organs, including the lungs, liver, and vascular system. A level of expression of genes involved in cell cycle progression and apoptosis may be induced by AgNP. The induction of ROS (reactive oxygen species), oxidative stress and DNA damage include possible AgNP-induced toxicity mechanisms.

In this paper the antimicrobial properties of wood–resin composites with the addition of silver nanoparticles solution (AgNPs) are presented. Moreover, the influence of the presence of AgNPs on the mechanical and thermomechanical properties as well as gloss of the obtained composites was investigated.

## 2. Materials and Methods

### 2.1. Chemicals

The unsaturated polyester resin used for composite preparation is a highly reactive orthophthalic resin of bluish-green color with enhanced chemical resistance properties and high strength parameters (LERG, Pustków, Poland). It was based on recycled poly(ethylene terephthalate) (PET). Luperox DHD-9 (2-butanone peroxide solution) (Sigma Aldrich, St. Louis, MO, USA) and a 4% polymeric cobalt solution (Department of Polymer Chemistry, UMCS Lublin, Poland) were used for its curing.

A virgin wood from spruce and fir commercial softwood JELUXYL WEHO (JELU-WERK, Rosenberg-Ludwigsmühle, Rosenberg, Germany) was used as raw material for modification of the unsaturated polyester resin. The wood particles were characterized by the following technical parameters: pH = 5.5, light yellow color and Alpine air sieve fraction: 75 μm (~35%), 100 μm (~20%) and 180 μm (traces). The manufacturer also declares that the moisture content is about 10%.

**Figure 1:**
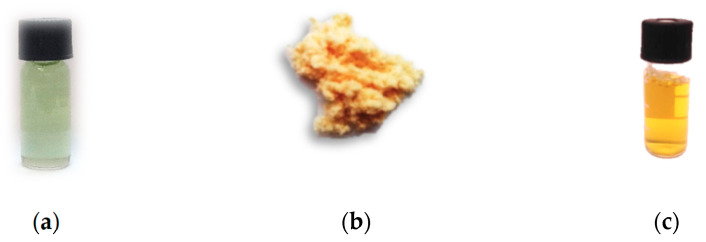
Images of components used in synthesis: (**a**) unsaturated polyester resin; (**b**) softwood flour; (**c**) silver nanoparticles concentrate.

Aqueous solutions of silver nanoparticles (AgNPs) NL-100 aqua (NANOLAB, Katowice, Poland) with antibacterial properties were used. Images of all components used in synthesis are presented at Figure 1.

### 2.2. Preparation of Composites

A detailed description of the synthetic method and the curing procedure was described previously [20]. The wood flour (WF) or the extract of silver nanoparticles in the aqueous solution (AgNPs) was added to pre-accelerated resin with 1.1 wt% of Luperox and 0.25 wt% of 4% polymeric cobalt solution, while mixing. All ingredient amounts were calculated for the pure resin. The prepared mixtures were poured into Petri dishes (50 mm diameter). Curing was conducted in these dishes at room temperature for 24 h and then at 80 °C for 10 h for additional post-curing. The same procedure was applied for the starting resin and their composites with WF or AgNPs. The compositions of the prepared composites are presented in Table 1.

### 2.3. Research Methods

#### 2.3.1. Preparation of Composite Samples

To study properties of the prepared composites, the samples of suitable sizes were prepared. The CNC milling machine MFG 8037P (Ergwind, Poland) was used for the preparation of samples in the shape of a cuboid (10 mm × 10 mm × 2.5 mm) for antibacterial tests, whereas for mechanical and thermomechanical studies molds of cuboid shapes with the dimensions 80 mm × 10 mm × 4 mm and 65 mm × 10 mm × 4 mm were applied.

#### 2.3.2. Accelerated Aging Test

Accelerated aging tests were performed using a Xenon Arc Lamp Atlas Xenotest Alpha + simulator (Chicago, IL, USA). The source of irradiation in the test chamber was a xenon lamp, emitting radiation similar to natural sunlight. Accelerating aging was carried out for 1000 h according to the standard EN ISO 4892-2:2013 [21]. The parameters imitated typical outdoor weather conditions to which materials can be exposed: irradiance 60 W m^−2^, daylight filter system, chamber temperature 38 °C, black standard temperature 65 °C, and relative humidity of 50%, spraying switch-on periods (18 min of spraying and 102 min of rest). 

#### 2.3.3. Mechanical and Thermomechanical Properties

Mechanical and thermomechanical properties were determined using a mechanical testing machine, a dynamic mechanical analyzer and a hardness tester.

The mechanical properties of the composites were determined based on the ZwickRoell Z010 mechanical testing machine from ZWICK GmbH Co (Ulm, Germany). Determination of mechanical properties was based on the three-point bending test, where the samples of 80 mm × 10 mm × 4 mm diameter were used with a span of 64 mm between the supports. The bending speed was 5 mm min^−1^. The test procedure was in accordance with the standard EN ISO 178:2019 [22]. Finally, the arithmetic averaging of five measurements was taken both before and after the accelerating aging test samples. 

Using a GYZJ 934-1 Barcol hardness tester from the Barber-Colman Company (Loves Park, IL, USA) the values of composite hardness were determined. The measurements were made according to the standard ASTM D2583 [23]. The final result was the average of ten measurements for the samples before and after the accelerated aging test.

All data were subjected to analysis of variance using Origin 8 (OriginLab, Northampton, MA, USA) applications. One-way analysis of variance (one-way ANOVA) was used to detect significant differences among the tested mechanical parameters (flexural strength and hardness) depending on the wood flour or silver nanoparticles content.

To determine thermomechanical properties of the prepared composites, the Q800 Dynamic Mechanical Analyzer (DMA) from TA Instruments (New Castle, NY, USA) equipped with a dual-cantilever device was used. Suitably prepared samples with the dimensions of 65 mm × 10 mm × 4 mm were tested. A temperature scanning from 0 to 200 °C was made with a constant heating rate of 3 °C min^−1^ at a sinusoidal strain with an amplitude of 10 µm and frequency 1 Hz. The test procedure was in accordance with the standard EN ISO 6721-1:2019 [24]. From the obtained data the glass-transition temperature, mechanical loss factor, values of storage modulus and loss modulus were determined. According to the standard [24] and due to the fact that the analysis was carried out over a wide range of temperatures, only one specimen before and after the accelerated aging test was measured.

#### 2.3.4. Determination of Gloss

Measurements of samples’ gloss were made using a Zehntner ZGM 1110 triple-angle gloss meter from Zehntner GmbH Testing Instruments (Sissach, Switzerland). This device operates simultaneously in one of three geometric units in which the angles 20°, 60° and 85° correspond from a high-gloss to matte surface (standard gloss: 20° (86.8 GU), 60° (93.4 GU) and 85° (99.7 GU)). These determinations were made according to the standard ASTM D2457 [25]. The final result was the mean value of ten measurements for the samples before and after the accelerated aging.

#### 2.3.5. Bacterial Strains

All tested samples were analyzed for their anti-biofilm potency in contact with aerobic Gram-positive *Staphylococcus aureus* ATCC 25923 and *Staphylococcus epidermidis* ATCC 12228; and aerobic Gram-negative *Escherichia coli* ATCC 25992 and *Pseudomonas aeruginosa* ATCC 27853. In the microbiological assay we used Mueller–Hinton broth (MH-broth) (BioMaxima, Lublin, Poland). After 24 h bacterial growth (at 37 °C) on agar, an inoculum in 5 mL of 0.9% was prepared, obtaining a density 0.5 McFarland (1.5 × 10^8^ CFU mL^−1^ (CFU: colony forming unit)).

#### 2.3.6. Anti-Biofilm Activity of Tested Materials

Seeding of materials with bacterial strains for biofilm formation determination was made to visualize viability of the biofilm structure [26]. The materials samples (unmodified one as a control (1); modified materials (2, 3, 4, 5, 6, 7, 8)) were washed in ethanol (2000 μL) and M-H broth (2000 μL) and moved to the bottoms of fresh 12-well polystyrene plates (NEST Biotechnology, Wuxi, Jiangsu, China). Then, these materials were covered with 2000 μL of M-H broth. Finally, 6.4 μL of tested bacteria inoculum (1.5 × 10^8^ CFU mL^−1^) was added. Wells with broth alone were also included in the experiment as a control for the sterility of the experiment. Thus, the obtained plates with material, broth and inoculum were incubated twice as long (48 h, 37 °C) to allow bacterial plankton cells to eventually attach to the material surface and form colonies and biofilm. The tests were performed using three replicates.

Then, the tested material was taken out into a fresh 12-well dish. It was then washed gently with 2 mL of 0.9% NaCl to eliminate loosely adhered planktonic cells of bacteria. The materials obtained in this way were subjected to a biofilm test using the confocal laser scanning microscopy technique (CLSM).

#### 2.3.7. Confocal Laser Scanning Microscopy—Biofilm Visualization

Using the procedure of double fluorescence staining of dead and living bacterial cells, the presence, architecture and structure of the viability of the biofilm can be demonstrated. In this assay, the Viability/Cytotoxicity Assay kit for Bacteria LIVE/DEAD Cells (Biotium, Fremont, CA, USA) was used [26,27]. After 48 h incubation with the tested strain, the biomaterials were moved to new plates and covered with 500 µL of 0.9% NaCl and live/dead dye (prepared by mixing 1 µL of DMAO with 1 µL of EthD-III in 8 µL of 0.9% NaCl). Five microliters live/dead dye solution obtained in such a way and 500 µL of PBS were added to each well containing the tested biomaterial. The stained biofilm was visualized after 15 min of dark incubation of the material with the dye in 0.9% NaCl. The whole material area was inspected to verify the presence of biofilms, then the most representative location (3180 µm × 3180 µm) was scanned for the experiment. The images were acquired using a confocal laser scanning microscope Olympus IX81 (Olympus, Tokyo, Japan) equipped with Olympus Fluoview FV1000 scanning head by Olympus Fluoview ver. 4.2c software (Olympus, Tokyo, Japan). Pictures were visualized with Imaris ver. 7.2.3 software (Oxford Instruments, Abingdon, UK). 

## 3. Results and Discussion

Generally, unsaturated polyester resins as cross-linked polymers are treated to resist biodegradation [28,29]. In the case of unsaturated polyester resins, there is no case when biological agents such as bacteria, fungi and their enzymes destroy the polymer; thus, its original form disappears. In order to increase the susceptibility of resins to microorganisms, many authors propose the use of biodegradable fillers [30,31,32]. That is why in our research resin composites with wood flour and composites with the addition of colloidal silver were used. The unsaturated polyester resin without any additives was used as a reference material.

In Table 2, Table 3 and Table 4 the results of mechanical and thermomechanical studies of these composites before and after the accelerating aging test are presented. It was assumed that the addition of wood flour into the resin would facilitate the existence of microorganisms on the surface. The data for the unmodified resin (sample 1) showed that after aging, the flexural strength decreased, whereas the hardness and mechanical loss factor significantly increased. This is related to additional hardening of the resin, which is also confirmed by the value of full width at half-maximum (FWHM). For the composites with wood flour (samples 2–4), a decrease in flexural strength was visible before and after the accelerating aging studies. The larger the wood content, the smaller their flexural strength is. A similar tendency can be observed for the values of mechanical loss factor. In turn, hardness increased after the wood was incorporated into the resin. It was particularly visible for the samples before aging. After aging, the composites lost their hardness. Even with the slightest addition of wood, they did not achieve hardness similar to that of the pure resin. Full widths at half-maximum for this series of composites became narrower [20]. Samples 5–7 were obtained by adding the colloidal solution of nanosilver into the resin. These samples were prepared to create materials completely immune to the action of bacteria. For these samples a significant increase in flexural strength before aging can be observed. After aging, its values decreased with increasing silver content. Similarly, the hardness of the obtained composites was much higher than that for pure resin. Their hardness increased evidently after aging. The mechanical loss modulus and full widths at half-maximum decreased insignificantly with the increasing content of silver. After aging, their values were lower. Sample 8 contained both wood and silver. Its flexural strength and mechanical loss factor were much lower compared to those of the pure resin, but similar to those of the wood composites. Due to the presence of silver it had greater hardness. It is also characterized by increased heterogeneity (FWHM). 

It seems that the lack of chemical binding between the inorganic silver nanoparticles and resin itself is the main cause of decreasing the mechanical properties. However, samples 5–8 can be treated as nanocomposites. The properties of such materials not only depend on those of their components but also crucially on their interfacial and morphological characteristics [33]. 

Analysis of variance showed that the values of flexural strength and Barcol hardness for samples with different percentages of the wood flour before and after accelerated aging were statistically significant, which was confirmed using one-way analysis of variance (ANOVA) at the significance level *p* < 0.05. The same observations occurred for composites with different percentages of silver nanoparticles.

**Figure 2:**
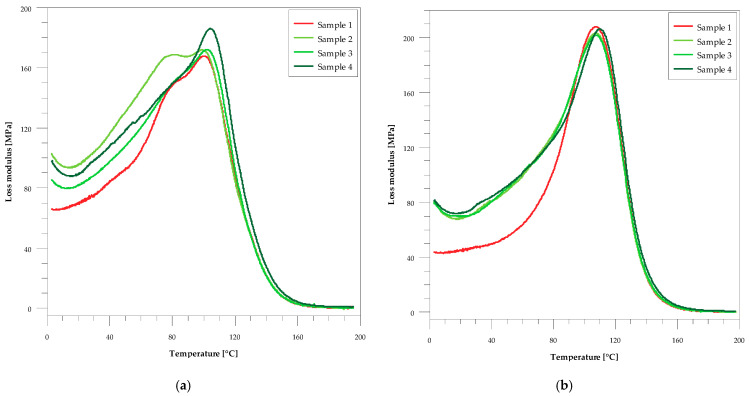
The temperature-dependent graph of loss modulus (E″) for samples with wood flour. (**a**) Before the accelerated aging test and (**b**) after the accelerated aging test.

**Figure 3:**
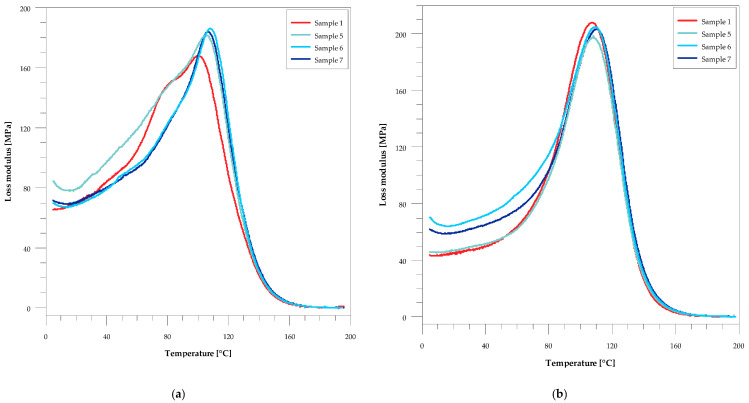
The temperature-dependent graph of loss modulus (E″) for samples with silver nanoparticles. (**a**) Before the accelerated aging test and (**b**) after the accelerated aging test.

In Table 3 the comparison of glass transition and storage modulus values for the composites before and after the accelerating aging test is presented. From these data one can see that, regardless of the method of glass transition temperature determination, for all the studied samples they became larger after the aging. The addition of both wood flour and silver caused an increase in T_g_. Increasing the wood content of the sample increased the transition temperature from 101 to 105 °C before aging and from 108 to 110 °C after aging. On the other hand, with the increase in the amount of silver nanoparticles in the material, an increase in T_g_ was observed from 106 to 109 °C before aging and from 108 to 111 °C after aging (Figure 2 and Figure 3). In this Table also the values of storage modulus in the glassy and rubbery regions, characterizing viscoelastic behavior of the sample, are presented. In the glassy region its values decreased slightly with the increase in filler content before aging. After aging, an insignificant increase in its value can be observed, especially for the samples with silver. In the rubbery region all samples had a greater storage modulus compared to that of the original resin before aging. It is particularly noticeable for the samples with the increasing amounts of wood flour. After aging, its values decreased for all samples except the unmodified resin. 

As can be seen from Figure 2 and Figure 3, the peaks in the loss modulus curves before ag-ing are asymmetric, and two glass transition temperatures can be determined from their shape. This indicates some inhomogeneity in these samples. Generally, the aging process of these composites resulted in improving homogeneity, which can be seen in both the FWHM values and the shape of the loss modulus curves.

The incorporation of silver nanoparticles has a beneficial effect on the mechanical and thermomechanical properties of the unsaturated polyester resin. In the case of composites, both before and after accelerated aging, the addition of AgNP caused an increase in the hardness of the glass transition temperatures compared to the original resin. 

Table 4 presents the results of gloss measurements. According to Zhao et al. [34], gloss is defined as the specular reflection ability of the material surface under a particular standard source and at a certain angle of incidence. This is an important parameter characterizing the surface optical properties of different materials. Gloss determination enables the evaluation of changes taking place on the surface of resins after aging [20]. Gloss is reduced when cracks and pitting appear due to the surface exposition to accelerated weathering conditions. This primary destruction can become a source of sample degradation. From the data in Table 4 one can see that before aging, all the obtained composites can be treated as high-gloss materials. Their values with 60° geometry, which is typically used, are much greater than 70 GU. For very high gloss materials such as unsaturated polyester resins, an angle of 20° is more recommended. For measurements at 20° the case is similar. In fact, a deterioration in gloss as the wood content increases and improvement in gloss with an increase in the silver content can be observed. After aging, the gloss values at 20° for the samples with wood decreased significantly. However, the samples with nanosilver still had a gloss above 70 GU. This indicates that the addition of AgNPs to the resin (samples 5–7) allows to obtain a high-gloss material after accelerated weathering. Similar properties indicate the wood–resin composite with silver nanoparticles (sample 8). Generally, it was found that AgNPs improved the gloss of the composites.

**Figure 4:**
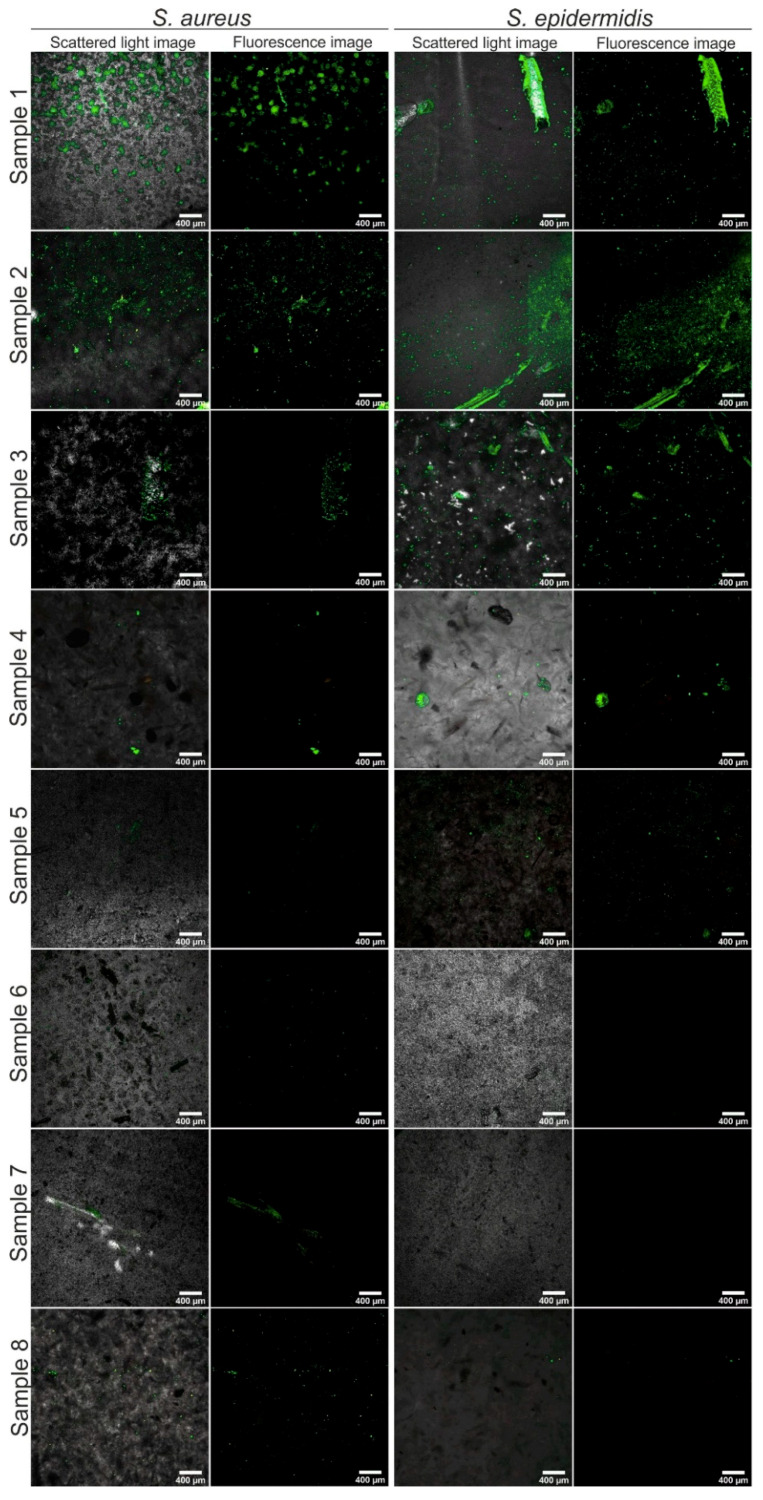
Confocal laser scanning microscope CLSM images showing Gram-positive strains biofilm formation (after 48 h of incubation) on tested materials: 1, control unmodified; 2–8, modified materials; magnification 40×; scale bar = 400 µm.

**Figure 5:**
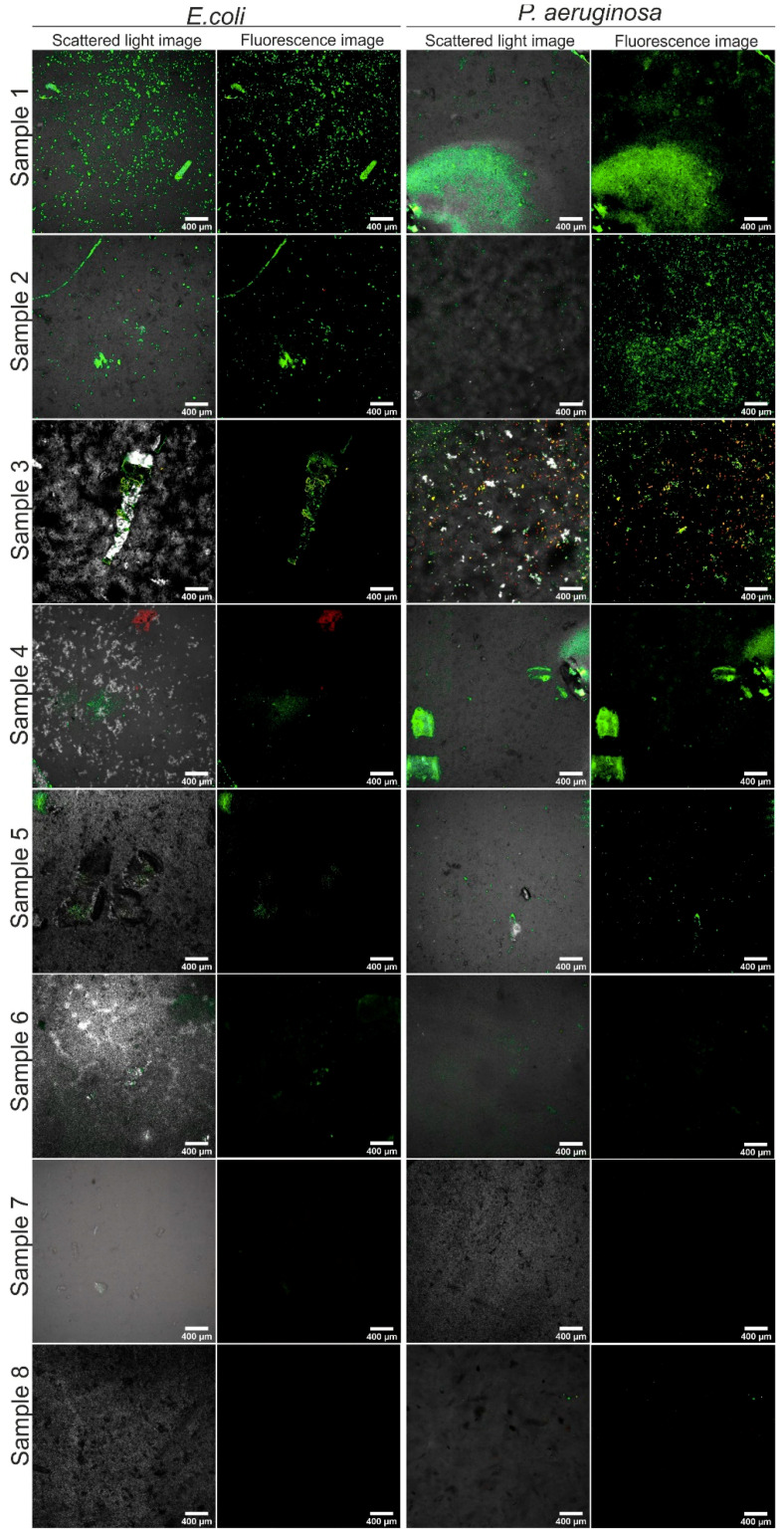
Confocal laser scanning microscope CLSM images showing Gram-negative strains biofilm formation (after 48 h of incubation) on tested materials: 1, control unmodified; 2–8, modified materials; magnification 40×; scale bar = 400 µm.

The effect of resin modifications on the power of biofilm formation was also tested. For this purpose, Gram-positive (Figure 4) and Gram-negative (Figure 5) strains were used. They were incubated with the materials long enough to allow the transformation of planktonic forms into an organized biofilm on the surface of the tested materials. Then, after washing away loose planktonic forms the samples were stained and imaged in a confocal microscope.

We noted that all tested strains (Gram-positive *Staphylococcus aureus* ATCC 25923, *Staphylococcus epidermidis* ATCC 12228; and aerobic Gram-negative *Escherichia coli* ATCC 25992, *Pseudomonas aeruginosa* ATCC 27853) could form biofilm architecture on the control unmodified material (sample 1). Green live bacterial cells merged into the forms and the groups that populated a large part of the control material. This is a completely unexpected result.

Whereas, on samples 2 and 3 surfaces a thin layer of biofilm was visible, regardless of the bacterial strain. Such live green colonies were uneven and formed wrinkles of the material. Material 4 showed on its surface a few single colonies of living cells with a thin layer of red dead bacterial cells. In sample 4 these bacteria also accumulate at the wrinkles. 

Importantly, the data presented in Figure 4 and Figure 5 indicate clearly that the composites modified with silver compounds (**5**–**8**) were completely resistant to biofilm formation under the tested conditions. Thus, materials 5–8 were resistant to biofilm formation across a broad spectrum of both Gram-negative and Gram-positive strains, regardless of the additional chemical components. 

Certainly, silver ions inhibit the viability of bacteria by bacterial membrane damage [35]. Many authors show use of silver nanoparticles as a beneficial way to prevention of infection [36,37,38,39]. Our observations are confirmed by the authors pointing to the anti-biofilm nature of the materials modified with silver-nanoparticles (AgNP). Significant reduction in biofilm density and increase in dead bacterial cells (especially *E. coli* and *P. aeruginosa*) on AgNP-modified materials, associated with destabilization of the bacterial membrane, was observed by Singh et al. [40]. Similar anti-biofilm potentials of AgNP structures were noticed by Hussain et al. and Pal et al. [41,42].

Sanyasi et al. reported that their AgNP inhibited biofilm formation, which notably caused bacterial membrane damage [35]. The adhesion of living cells, both *Eukaryotic* or *Prokaryotic*, to abiotic surfaces may be influenced by the chemical structure of these materials, size, shape, capping layer, functional groups and smoothness/wrinkles. The features of the composition and surface type of the material affect initial interactions and, consequently, adhesion to the cell membrane.

Many authors point to the possibility of using colloidal or ions or silver nanoparticles in vivo, where other antibiotic therapies are not effective. However, studies showing the optimal (safe for the patient and antibacterial-effective) doses of such silver compounds are still needed to obtain a therapeutic effect against Gram-negative and Gram-positive bacteria [43].

Therefore, it seems to be advantageous to obtain materials with the addition of silver compounds, which would enable their applicability not only in the household but also in pharmacy, analytical and medical as well as food industries.

Biocides, such as our silver-modified materials, have a broad spectrum of activity; they can inhibit the viability of not only microbes but also other organisms, including eukaryotic cells [43]. Thus, in the next step of our research we will study cytotoxicity against normal eukaryotic fibroblasts lines, to possess information about the safety of using our materials in close contact with human skin.

## 4. Conclusions

As it was assumed, Gram-negative and Gram-positive bacteria colonize mainly in various wrinkles as well as cracks on the composites with wood flour. In turn, the resins modified with silver nanoparticles (AgNP) were completely resistant to biofilm formation. Importantly, the obtained results indicate that bacterial strains can colonize the surface of the cross-linked unsaturated polyester resin. This can change the current views on the disposal of products made of unsaturated polyester resins. 

Favorable effects of silver nanoparticles incorporation on mechanical and thermomechanical properties as well as gloss of unsaturated polyester resin were observed. For the composites before and after accelerated aging, the addition of AgNPs caused an increase in flexural strength and hardness compared to those of the original resin. A significant impact on the glass transition temperatures was observed. 

In the case of the composites with wood flour, deterioration of mechanical properties was observed. As the wood flour content increased, they became brittle, and their gloss diminished. 

As was expected, silver nanoparticles modification promoted antimicrobial activity of the wood–resin composites in contact with pathogens such as *S. aureus*, *S. epidermidis*, *E. coli* and *P. aeruginosa*. The materials characterized by such properties are promising for medical field applications.

Importantly, the addition of nanosilver caused the death of bacteria not only on the surface of the unsaturated polyester resin but also on the surface of the composite with biofiller. This may be an indication for manufacturers of devices for medical purposes. In order to reduce the cost and consumption of the resin, the addition of biofillers can be put into practice, provided that the product will contain nanosilver.

## Figures and Tables

**Table 1:** The compositions of the prepared composites.

Sample	UPR (wt%)	WF (wt%)	AgNPs aq. sol. (wt%)
1	100.0	-	-
2	99.0	1.0	-
3	98.0	2.0	-
4	95.0	5.0	-
5	99.8	-	0.2
6	99.5	-	0.5
7	99.0	-	1.0
8	94.0	5.0	1.0

**Table 2:** Mechanical and thermomechanical data for the studied composite samples before and after the accelerated aging test.

Sample	Flexural Strength, σ_f_ (MPa)		Barcol Hardness, HBa (°B)		Mechanical Loss Factor, Tan δ_max_		Full Width at Half Maximum, FWHM (°C)	
	before	after	before	after	before	after	before	after
1	108.19 ± 3.77	69.26 ± 4.35	36.0 ± 1.0	56.4 ± 0.4	0.4579	0.4839	46.1	39.7
2	81.96 ± 2.71	50.03 ± 3.33	39.5 ± 0.5	55.7 ± 0.7	0.4419	0.4510	45.9	39.6
3	76.83 ± 2.65	40.95 ± 4.27	40.3 ± 0.5	54.1 ± 0.6	0.4368	0.4397	43.4	40.0
4	74.61 ± 1.48	39.43 ± 4.39	45.2 ± 0.7	51.3 ± 0.5	0.4227	0.4131	42.4	38.5
5	119.05 ± 2.65	72.31 ± 4.82	48.8 ± 0.8	57.0 ± 1.0	0.4543	0.4575	40.6	41.9
6	121.81 ± 1.97	70.71 ± 4.45	49.2 ± 0.2	57.5 ± 0.5	0.4513	0.4427	42.0	41.2
7	128.74 ± 1.27	70.03 ± 3.69	53.3 ± 0.3	58.8 ± 0.8	0.4479	0.4408	42.7	41.1
8	76.69 ± 1.90	51.95 ± 3.51	55.0 ± 1.0	60.6 ± 0.6	0.3383	0.3328	47.6	46.2

**Table 3:** Glass transition temperature and storage modulus in the glassy and rubbery regions (from the storage modulus curve) for the studied composites before and after the accelerated aging test.

Sample	Glass-Transition Temperature, T_g_ (°C)				Storage Modulus, E′			
	From tan δ		From Loss Modulus Curve, E″		Glassy, E′(20 °C) (GPa)		Rubbery, E′(180 °C) (MPa)	
	before	after	before	after	before	after	before	after
1	127	132	100	107	2.91	2.81	24.06	25.24
2	126	131	101	108	3.17	2.78	31.45	23.78
3	126	131	103	108	2.94	2.81	34.68	28.50
4	126	130	105	110	2.86	2.90	49.08	40.04
5	128	131	106	108	2.79	2.73	27.13	22.05
6	129	131	108	110	2.78	2.83	27.53	23.33
7	130	132	109	111	2.75	2.86	27.75	25.75
8	126	129	104	107	2.77	2.83	46.11	41.20

**Table 4:** Gloss measurement data of the studied composites before and after the accelerated aging test.

Sample	Gloss (GU)					
	20°	60°	85°	20°	60°	85°
	before Aging			after Aging		
1	101.3	112.4	100.4	30.6	50.2	81.1
2	96.8	110.4	98.5	63.3	92.2	96.0
3	96.4	101.8	99.2	57.5	84.0	90.3
4	76.8	95.3	96.1	55.3	81.0	87.6
5	113.3	121.8	99.9	85.9	97.3	98.1
6	128.3	133.9	102.9	83.9	96.8	98.1
7	107.1	121.1	102.0	83.4	91.6	94.6
8	94.7	100.4	100.3	73.5	90.2	95.5

## Data Availability

Data sharing is not applicable to this article.
